# An Efficient Compression Method of Underwater Acoustic Sensor Signals for Underwater Surveillance

**DOI:** 10.3390/s22093415

**Published:** 2022-04-29

**Authors:** Yong Guk Kim, Dong Gwan Kim, Kyucheol Kim, Chang-Ho Choi, Nam In Park, Hong Kook Kim

**Affiliations:** 1 Gwangju Institute of Science and Technology, School of Electrical Engineering and Computer Science, Gwangju 61005, Korea; bestkyg@gist.ac.kr; 2 LIG Nex1, Maritime R&D Center, Seongnam-si 16911, Korea; donggwan.kim@lignex1.com (D.G.K.); kyucheol.kim@lignex1.com (K.K.); changho.choi@lignex1.com (C.-H.C.); 3 National Forensic Service, Digital Analysis Division, Wonju-si 26460, Korea; naminpark@korea.kr

**Keywords:** underwater acoustic sensor, data compression, sensor signal compression, underwater surveillance

## Abstract

In this paper, we propose a new compression method using underwater acoustic sensor signals for underwater surveillance. Generally, sonar applications that are used for surveillance or ocean monitoring are composed of many underwater acoustic sensors to detect significant sources of sound. It is necessary to apply compression methods to the acquired sensor signals due to data processing and storage resource limitations. In addition, depending on the purposes of the operation and the characteristics of the operating environment, it may also be necessary to apply compression methods of low complexity. Accordingly, in this research, a low-complexity and nearly lossless compression method for underwater acoustic sensor signals is proposed. In the design of the proposed method, we adopt the concepts of quadrature mirror filter (QMF)-based sub-band splitting and linear predictive coding, and we attempt to analyze an entropy coding technique suitable for underwater sensor signals. The experiments show that the proposed method achieves better performance in terms of compression ratio and processing time than popular or standardized lossless compression techniques. It is also shown that the compression ratio of the proposed method is almost the same as that of SHORTEN with a 10-bit maximum mode, and both methods achieve a similar peak signal-to-noise ratio (PSNR) and structural similarity (SSIM) index on average.

## 1. Introduction

Research on sonar technology for underwater surveillance has been ongoing since World War II. In recent years, sonar technology for military purposes, as well as commercial and scientific research, has been actively developed using underwater sensors. Sonar can be classified into the following two basic types: active and passive sonar. Both types use underwater acoustic sensors called projectors and hydrophones for underwater sources and receivers, respectively. In most active sonar systems, the same transducers can be used as both projectors and hydrophones. On the other hand, passive sonar systems for search, surveillance, or various noise monitoring purposes use only hydrophones. The hydrophones detect the pressure variations in the acoustic signals and noise in the water and produce an output voltage proportional to the pressure [1]. Thus, in this paper, we focus on the acoustic signal obtained through the hydrophones for the passive sonar system mentioned above, and we refer to a sensor as a hydrophone.

To date, various sensors for underwater surveillance have been reported [1,2] and are commonly classified into the following several categories: piezoelectric sensors [1], which use piezoelectric effects; magnetostrictive sensors [1], which use the magnetostriction of the Earth’s magnetic quality due to the target’s magnetic properties; fiber-optic sensors, which use optical fibers as the sensing element. In a real application, piezoelectric sensors are generally used as underwater acoustic detection sensors due to their low cost and high efficiency for transduction [1]. Consequently, in this study, we deal with the applications of piezoelectric sensor-based systems.

**Figure 1:**
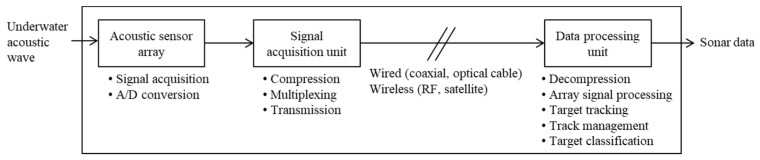
General structure of an underwater surveillance system based on acoustic sonars.

The goal of sonar systems for underwater surveillance is reliable, long-range detection. Figure 1 shows the general structure of an underwater surveillance system based on acoustic sonars. The main function required of a sonar system is to effectively eliminate interference and ambient noise. The underwater acoustic sensors are assembled as arrays to improve the response of the array, thereby increasing the signal-to-noise ratio (SNR) and allowing the determination of the direction of a signal source [3,4]. The most common sonars today comprise an array of hydrophones combined with appropriate time delays to form beams in the desired direction or multiple directions. In particular, passive sonar systems rely very much on the ability of their sensors to capture the sound arriving from different directions. Typically, hydrophones are arranged in arrays for detection from all directions. The hydrophone array may be linear, planar, circular, or cylindrical.

The number and arrangement of the acoustic sensors constituting the sensor array are designed according to the frequency band to be detected, and the array gain performance to be achieved [4,5] through array signal processing. Since the sensor array consists of tens to hundreds of sensors to obtain high array gains [1], the amount of sensor data acquired every second is enormous, and the amount of operating power consumed by constant monitoring is large. Therefore, on a fixed platform on which power and communication lines are connected to the land, or on a mobile platform such as a surface ship or submarine with sufficient power supply, the power and communication resources for sensor operation and signal transmission should be sufficiently supplied.

However, a low-power design with a low computational load is essential for unmanned platforms such as autonomous underwater vehicles (AUV), unmanned underwater vehicles (UUV), and offshore buoys or underwater wireless sensor networks with limited power and communication resources [6]. It is also necessary to reduce the amount of data required to communicate the acquired signal information through wireless communication or satellite because these communication devices also consume electrical power for transmission [6,7]. In addition, an underwater acoustic network having a narrow acoustic transmission bandwidth uses an acoustic sensor for data transmission in order to reduce the amount of data required for the transmission [8]. Large sensor networks need a lot of power to transmit data. Therefore, data compression is unavoidable to reduce the power consumption and amount of information [9]. In addition, it is necessary to reduce the amount of data in terms of the cost of securing hardware resources to acquire and store acoustic data in a system that operates without interruption for a long period. Therefore, a low-complexity source compression technique should be applied to the sensor signals for real-time operation in an environment with limited power and bandwidth.

Recently, various techniques for compressing high-quality multi-channel audio have been studied, and the standardization of compression techniques has also progressed [10,11]. However, until now, there have been relatively fewer studies on underwater sensor signal compression than on audio compression, and most of them were conducted on applications for marine noise monitoring [9,12]. In addition, most of the existing approaches for compressing underwater sound convert sound into their corresponding two-dimensional acoustic images, such as passive sonar gram or active sonar B-scan image, and then apply an image compression technique [13]. Only a few studies have focused on the raw sensor signal [14,15,16]. Furthermore, despite the increasing necessity of collecting and processing large amounts of underwater acoustic sensor data for data learning in artificial intelligence algorithms, there have been few attempts to deal with collecting and storing data [17,18].

Generally, audio compression can be categorized into lossy and lossless techniques depending on whether the original signal can be completely recovered or not [10,16]. Lossy compression techniques utilize human auditory perception to achieve a higher compression rate with some acceptable distortion than lossless compression techniques. In another category, near-lossless compression techniques increase the compression ratio by applying transformations of coding parameters with near-perfect reconstructions to maintain perceptual transparency [11,19]. While most audio compression technology utilizes human auditory perception to reduce bit rate [11], it is inefficient to directly apply audio compression technology to applications for underwater sensor signals due to differences in the sampling rates and the statistical characteristics of signals. The quantizer of lossy audio codecs employs the so-called psychoacoustic model to determine how many bits are allocated. However, since the discrete samples generated by a hydrophone also have natural temporal and spatial correlations, just like audio signals, these digital data are expected to be redundant [15]. For this reason, some studies have used predictive coding-based audio compression technology to compress underwater acoustic sensor signals [12,15,16].

The systems in active sonar applications, e.g., transducers and signal processing units, usually operate and detect acoustic ping signals over tens of kHz. However, the systems in passive sonar applications, e.g., hydrophones and signal processing units, detect the self-radiated noises from a ship’s or submarine’s machinery distributed in the lower frequency band within 2 kHz [4]. In particular, noise in the form of discrete tones, which is radiated from submarines, is weak compared with other noise components, such as unidirectional or directional broadband noise. These discrete tones are important for identifying ship information, and it is also important to compress this information without distortion. Therefore, a lossless or near-lossless encoding technique is required for the compression of sensor signals to minimize the deterioration of detection performance due to the loss of information.

Consequently, the aim of this paper is to provide an alternative compression method for underwater acoustic sensor signals. With the aid of recent research in audio compression, this paper proposes an efficient compression method for underwater acoustic sensor signals. The proposed compression method is based on a lossless compression scheme that minimizes the loss in the frequency band of interest in a low-complexity structure in order to minimize power consumption based on the scientific and engineering analysis of the characteristics of the underwater acoustic sensor signal.

The remainder of this paper is organized as follows: Section 2 briefly describes the characteristics of underwater sound. Section 3 presents the proposed sensor signal compression method. After that, Section 4 evaluates the performance of the proposed method using real sensor signals acquired from several different sensor arrays deployed in a real underwater environment by measuring the compression ratio and processing time. Finally, Section 5 concludes this paper.

## 2. Underwater Sound

This section explores the characteristics of underwater sound. The key question to ask, from our perspective, is which coding scheme is the best for an underwater acoustic sensor signal. To answer this, it is necessary to understand the characteristics of the underwater acoustic signal. We need to consider the relationship between noise sources and the frequency bands, in which the noise is generated. It is highly probable to apply a method of using different coding schemes for important and non-critical frequency bands.

The underwater acoustic sensor receives noise signals from various sources along with the noise signals from the target of interest. The potential sources of this noise are turbulence, shipping, wave action, thermal agitation, seismic events, rainfall, and marine animals [4]. The main sources of such noise are the following: earthquakes, marine life, ships, waves, or wind, and each of these factors has a different dominant band [3,4]. The characteristics of underwater noise were first studied by Knudsen et al. [20] during World War II and also studied by Wenz [21] and Urick [22].

According to [21,22], the frequency band in which shipping noise is dominant is from approximately 10 Hz to 1 kHz. The frequency band less than 10 Hz is the band affected by turbulence. For this reason, low-frequency acoustic sensors for detecting the self-noise of ships are generally designed and manufactured to receive acoustic signals within 2 kHz. Thermal, wave, and rain noise generate continuous frequency bands with Gaussian statistics. Shipping noise, however, contains both continuous noise from propeller cavitation and discrete noise from machinery and blade rate components [4]. Radiated noise types are divided into the following two general categories: broadband and narrowband noise [4]. The noise from ships is mainly composed of engine and propeller noise. In particular, the noise generated by the engine shows discrete tone noise characteristics in the frequency domain, and the noise generated by the propeller shows broadband noise characteristics. Unlike broadband noise, narrowband noise in the form of a discrete tone is an important factor in the detection and classification of ship targets. Some narrowband noises are hard to detect because the amplitude of background noise is much higher than that of narrowband noise and the directivity index (DI) of the array is lower at the lower frequency band [4]. In addition, the amplitude of a sinusoidal signal from a distant source fluctuates when the signal arrives at the receiver due to several propagation effects [22]. Furthermore, the broadband noise dominated by screw and flow noise tends to increase in proportion to the maneuvering speed of the vessel, and it is difficult to observe the narrowband noise due to the masking effect of the broadband noise generated at high speed. Therefore, it is important to apply a compression technique to minimize distortion and the loss of information about weak narrowband noise components.

In the case of a system for detecting submarines and submersible targets in the water, broadband noise from propellers may not occur when the target is submerged, and only narrowband noise caused by engines or air conditioners is generated. The frequency of this weak narrowband noise is distributed over less than 1 kHz. In addition, in the case of frequency bands less than 100 Hz, the broadband noise, harmonic components of the strong narrowband noise components (e.g., narrowband components corresponding to blade rate (BR), propeller shaft rate (PSR), and diesel firing rate) and electrical (60 Hz fundamental) noise components radiating from large ships are mainly distributed. Therefore, this frequency band is not a band of interest for the purpose of monitoring underwater targets.

**Figure 2:**
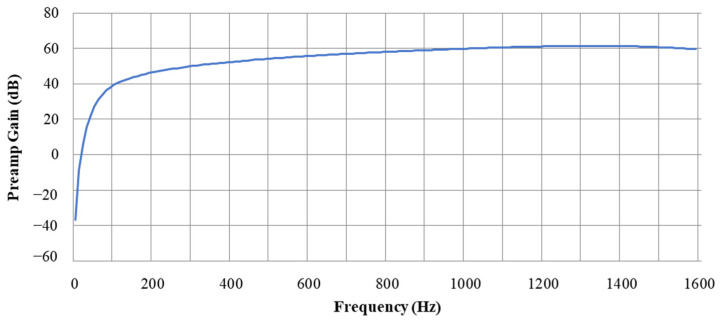
Preamp gain of an acoustic sensor reflecting underwater sound characteristics.

**Figure 3:**
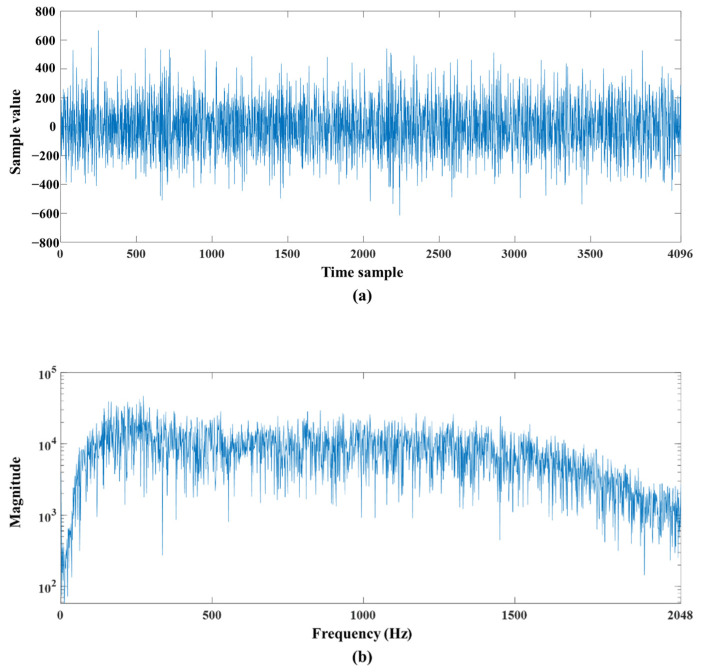
Examples of acquired sensor signals represented in (**a**) time domain and (**b**) frequency domain.

In connection with this characteristic, we propose a method based on the sub-band splitting approach in the next section. Figure 2 shows the preamp gain of the acoustic sensor designed by applying such underwater sound characteristics, and Figure 3 shows the output of sensors represented in the time domain and frequency domain. 

## 3. Proposed Method

This section describes the encoder and decoder structures of the proposed method that is based on a sub-band splitting and scalable structure to increase the coding efficiency. By independently encoding the separated sub-bands, the encoder prioritizes and transmits the low-frequency band in an environment where network bandwidth is limited, and the decoder can restore the received low-frequency band without loss. Thus, a scalable encoding function is provided.

### 3.1. Encoder

**Figure 4:**
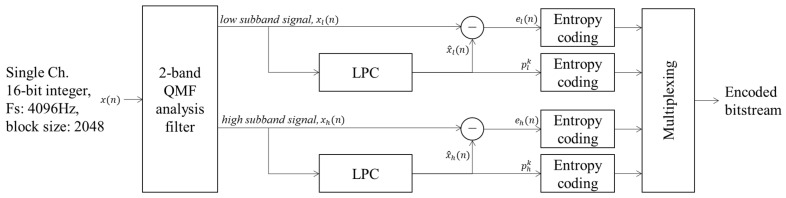
Block diagram of the encoder structure of the proposed method.

**Figure 5:**
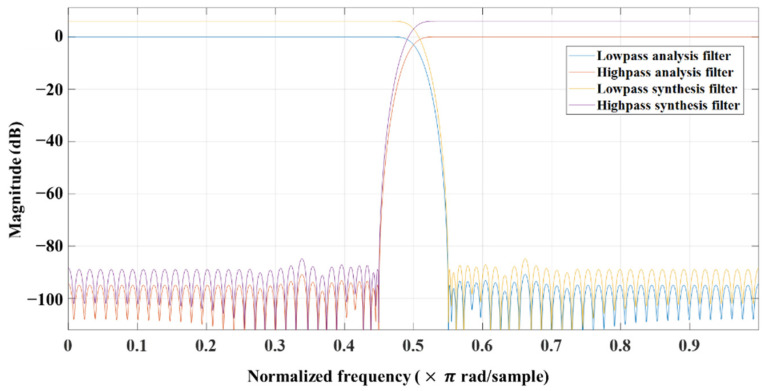
Magnitude responses of two-band QMF analysis and synthesis filters used in the proposed method.

Figure 4 shows the proposed encoder structure for the compression of acoustic sensor signals. As discussed in Section 2, passive sonar systems for underwater surveillance can detect frequency components up to 2 kHz, where ship noise is mainly distributed. Therefore, the sampling rate is usually set at 4096 Hz in an underwater surveillance system. As shown in the figure, the input signal is decomposed into two sub-bands by a filter bank, as in Moving Picture Experts Group (MPEG) audio. The two-band QMF analysis filterbank decomposes the input into high-frequency and low-frequency sub-bands, where each sub-band has half the bandwidth of the input [11,23]. In this paper, we design a two-band QMF analysis and synthesis filter with a finite-duration impulse response (FIR) filter with an order of 127. Figure 5 shows the magnitude responses of two-band QMF analysis and synthesis filters, respectively. Each low-band or high-band filter is applied to the input signal, and then the filtered signal is down-sampled by a factor of two. After that, each sub-band signal is quantized according to a suitable bit allocation rule using the fast Fourier transform (FFT) of the input signal and the psychoacoustic model [24]. However, in the proposed method, a sub-band analysis technique is performed for scalable coding, and the two-band quadrature mirror filter (QMF) analysis filter decomposes a signal into high- and low-frequency sub-bands. This analysis QMF filter achieves an almost perfect reconstruction [11].

The critical point of the proposed method is that it performs linear prediction-based lossless compression similar to an audio codec, except that the signal is split into two sub-bands in advance; thus, scalability is obtained through independent coding. In addition, the proposed method tries to find parameter values, such as coefficients for linear predictors and parameters for entropy encoders, that are suitable for underwater acoustic signals when performing compression for each sub-band.

In an environment where the communication bandwidth is extremely limited, a scalable structure is often used so that only important low sub-band signals are transmitted to the receiver. This is because shipping noise is relatively insignificant above 1 kHz, and noise below 1 kHz is likely to be dominated by discrete tones [3]. The high sub-band in which the signal corresponds to 1–2 kHz is a frequency component where singing or cavitation noise is generated by propellers, fluid noise caused by the maneuvering of a ship, etc., is present. Unlike in the low sub-band, it is necessary to analyze the broadband noise energy rather than the narrowband frequency value. The reason for preserving the high sub-band by performing compression without completely blocking the high-frequency band is that it preserves the broadband noise component in the low sea state [4]. In addition to cavitation and resonance noise (i.e., singing noise), a broadband noise component is often required for the separation of surface and underwater targets. Furthermore, this is a frequency band where modulation noise for the PSR and BR components can occur as discrete lines because these are used for the analysis of a target’s propulsion systems, such as propellers and shafts. Depending on the monitoring purpose, the high sub-band is not transmitted as mentioned above, or lossy compression is also applicable.

After the encoder finishes compression for each frame of each band, the prediction coefficients used for linear prediction, the information in the compressed frequency band, and the parameter values used for entropy coding are transmitted to the decoder as side information.

### 3.2. Decoder

As mentioned in Section 3.1, the encoder proposed in this paper transmits an acoustic signal separated into two sub-bands for each band independently, so that the decoder can independently decode only one sub-band. That is, the proposed encoder and decoder can perform scalable encoding and decoding, respectively. When the bandwidth of the transmission channel is extremely limited, the decoder of the receiver restores only the transmitted band independently and can be applied to target detection.

**Figure 6:**
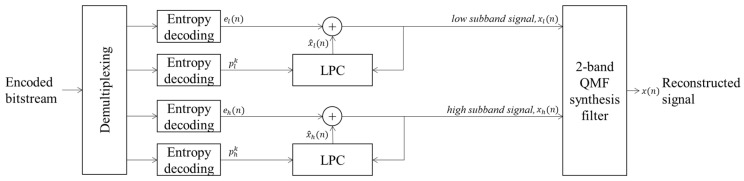
Block diagram of the decoder structure of the proposed method.

Figure 6 shows the decoder structure of the proposed method. As shown in the figure, the decoder checks whether a high-frequency sub-band is included in the encoded bitstream according to the current mode information. For each sub-band, entropy decoding is first performed on the linear prediction coefficients and residuals. Then, linear prediction is performed, and lossless restoration is finally carried out by adding the residuals. The entire band signal is reconstructed using the synthesis filter bank on the time domain signal reconstructed for each sub-band.

### 3.3. Linear Prediction

As mentioned above, low- and high-frequency sub-band signals are separately compressed losslessly by linear prediction with an entropy coder. Linear prediction coding (LPC) is commonly used for encoding speech and audio to predict the current sample using a linear combination of K immediate predecessors [15], and in recent years, it has emerged as a common and practical technique for lossless audio compression [25]. A finite impulse response linear predictor of the K-th order predicts the current audio sample, x(n), as follows:

(1) $$\hat{x}\left(n\right)=\sum_{k=1}^{K}a_{k}x\left(n-k\right)$$

where $a_{k}$ is the kth prediction coefficient. The prediction coefficients are estimated by using the autocorrelation method, as in MPEG4-audio lossless (ALS) [26]. Since the prediction coefficients are very sensitive to even small quantization errors [10], we first convert the prediction coefficients into partial correlation (PARCOR) coefficients by using the Levinson–Durbin algorithm. Then, PARCOR coefficients are quantized and multiplexed into the bitstream. The quantized PARCOR coefficients are converted back to the prediction coefficients to obtain the reconstructed signal, $\hat{x}\left(n\right)$, and the residual signal, e(n), is computed as follows:

(2) $$e\left(n\right)=x\left(n\right)-\hat{x}\left(n\right)$$

In low-bit-rate speech coding, an all-pole filter-type predictor is used, which is mostly implemented as a 10th order predictor [11]. In this paper, to find an appropriate LPC order for each sub-band signal, we measured and compared a compression ratio and the processing time of encoding by changing the LPC order from 1 to 30. The compression ratio is defined as follows [10]:

(3) $$C_{R}=\frac{Compressed\;file\;size}{Original\;file\;size}\times 100\;\left(\%\right)$$

and the processing time is calculated as follows:

(4) $$T_{proc}=T_{end}-T_{start}$$

where $T_{end}$ and $T_{start}$ denote the times measured at the start and end points of encoding, respectively.

**Figure 7:**
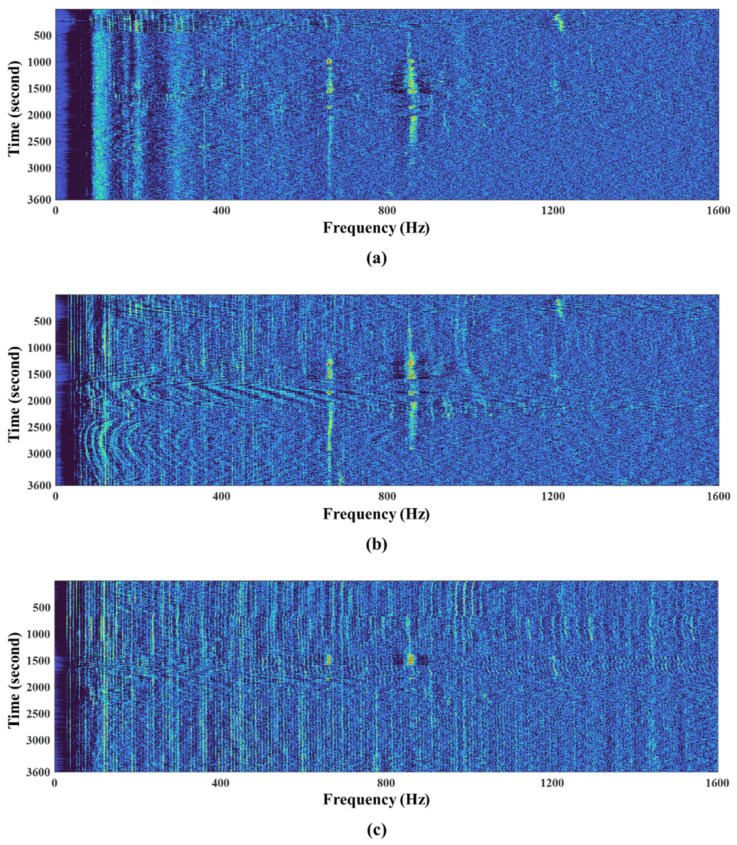
Illustration of LOFAR-grams of sensor signals from (**a**) sensor #1, (**b**) sensor #2, and (**c**) sensor #3.

To this end, raw signals for one hour were acquired from three different sensors operating at the same time and in the same coastal area, and they were uniformly quantized with 16 bits, where the sampling rate was also set to 4096 Hz. Note here that the three sensors were located about 1.6 km apart from each other. Figure 7 illustrates the signal low-frequency analysis recording (LOFAR)-gram for each case. The LOFAR-gram is a popular tool for analyzing narrowband signals in a passive sonar-based surveillance system by displaying the outputs from a selected sensor or beamformer in a frequency versus time format [3]. Therefore, a LOFAR-gram contains information for the classification and analysis of contact motion [3]. As shown in the figure, one vessel (vessel #1) passed near sensor #2 and sensor #3, and the other (vessel #2) passed near sensor #1. Vessel #1 radiated strong tonal noise at between 650 and 850 Hz, and vessel #2 also radiated strong tonal noise at 1.2 kHz. In addition, when the ship approached the closest point of approach (CPA) to the sensor, it could be observed that strong broadband noise was detected over the entire band along with the tonal component.

**Figure 8:**
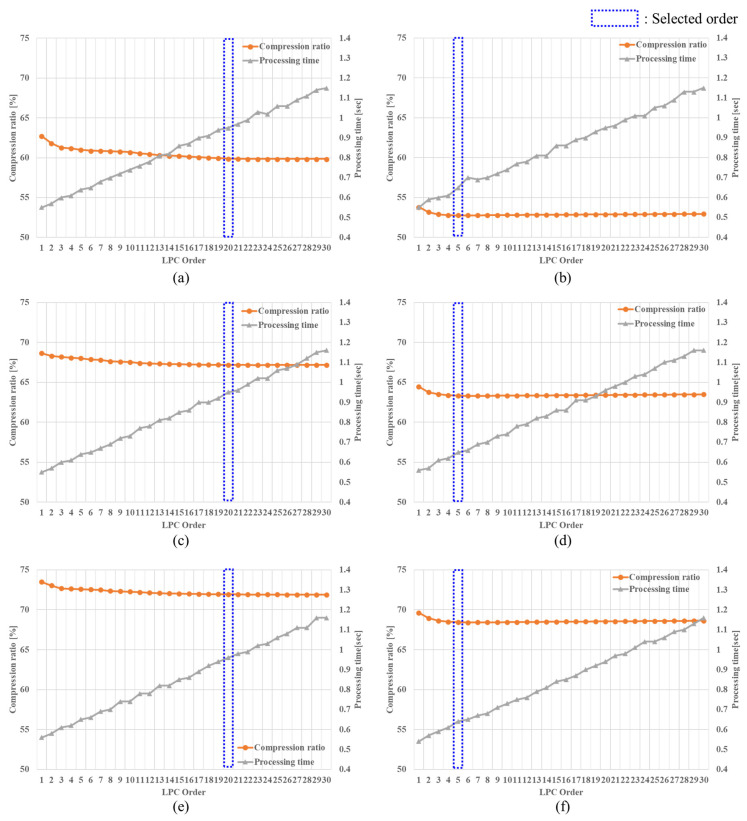
Comparison of the compression ratio and processing time according to different LPC orders: (**a**) sensor #1 (low sub-band), (**b**) sensor #1 (high sub-band), (**c**) sensor #2 (low sub-band), (**d**) sensor #2 (high sub-band), (**e**) sensor #3 (low sub-band), and (**f**) sensor #3 (high sub-band).

Figure 8 shows the measured result for the compression factor and processing time of the proposed method. It could be observed from the figure that the LPC analysis order for both low and high sub-band signals converged at a specific LPC order in view of the compression ratio. However, as expected, the processing time increased proportionally as the LPC order increased. Through this analysis, it was possible to specify the appropriate LPC order for the two sub-bands of the underwater acoustic signal. Consequently, we set the LPC order between 20 and 5 for the low and high sub-bands, respectively.

### 3.4. Entropy Coding

We employed Rice codes when coding the residual because Rice coding can be efficiently implemented in central processing units (CPUs) using bit shift and bit masking without the need for floating-point operations, which allows for extremely simple and fast encoding. Rice coding is used in lossless audio encoders such as SHORTEN [25], free lossless audio codec (FLAC) [10], and MPEG4-ALS [26]. It is known that Rice coding can provide short codes close in length to Huffman codes [27]. Robinson [25] observed that the distribution of the residual signal in LPC-based audio encoders could be closely modeled by a Laplacian or two-sided geometric distribution. However, the compression performance of the Rice encoding technique is most affected by the Rice parameter value, and thus many studies have been conducted to find the optimal Rice parameter for encoding an audio signal [27,28,29]. Robinson also proposed an optimal Rice parameter for a data sequence that follows a Laplacian distribution.

**Figure 9:**
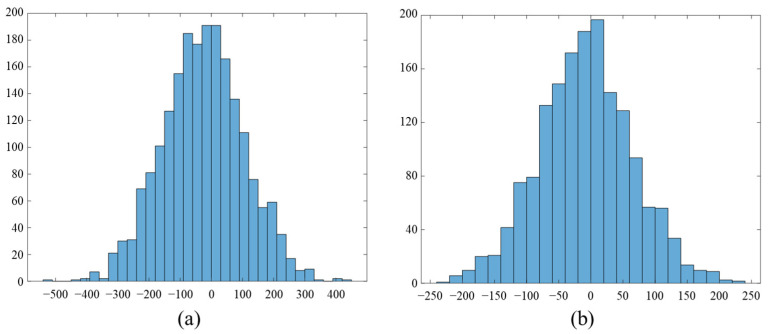
Illustration of the observed residual distributions of (**a**) low sub-band and (**b**) high sub-band signals.

Figure 9 shows the observed distribution of a residual computed from sensor #1 as a histogram. As shown in the figure, the distribution of the residual signal from LPC for underwater acoustic sensor signals can also be modeled by a Laplacian distribution. This implies that Rice coding is applicable to encoding underwater acoustic sensor signals. For this reason, in this study, we utilize an estimator for the optimal rice parameter that has already been used in MPEG-4 ALS and SHORTEN as follows:

(5) $$s=⎣log_{2}\mu_{n}+C⎦$$

where $\mu_{n}$ is the absolute mean of residuals ($\mu_{n}=\frac{1}{N}\sum_{i=1}^{n}\left|r_{i}\right|$), and C (= 0.97) is a constant. The Rice parameter, s, is estimated for each sub-block and transmitted along with the encoded residuals [30]. This is the same as in the existing MPEG-4 ALS method.

## 4. Performance Evaluation

To evaluate the performance of the proposed method, we measured the compression ratio of the proposed method using Equation (3), the processing time of compression, and the distortion of the sensor signal due to compression. To this end, sensor signals were acquired from five different actual underwater sensors operating in coastal areas. These sensor signals were acquired at the same time from the different sensors operating in the same coastal area as the sensors (sensors #1, #2, and #3) used for the analysis in Section 3.3. In other words, we used different sensors for performance evaluation from those used in the analysis. These five sensors were located 800 m apart from each other. Each sensor signal was recorded for one hour at a sampling rate of 4096 Hz, and they were uniformly quantized with 16-bit resolution. Table 1 summarizes the specifications of the acoustic sensor signals used for performance evaluation. Note here that the prediction orders for the proposed method were set between 20 and 5, respectively, for the low sub-band and high sub-band. In addition, a Rice parameter was estimated using Equation (5).

### 4.1. Compression Efficiency

In this evaluation, the compression ratio of the bitstream encoded by the proposed compression method was compared with that of the compression methods employed in MPEG-4 ALS [10,31], FLAC [10], and WavPack [10]. Table 2 shows a list of the compression methods compared in this study. In other words, MPEG-4 ALS was implemented with three different compression modes according to different LPC orders and entropy coding schemes, as shown in the first three rows of the table. In addition, FLAC and WavPack were each implemented with the following different modes: fast and high-quality mode.

Table 3 compares the compression ratios of the proposed compression method with those of seven different compression methods described in Table 2. As shown in the table, the proposed method provided the smallest compression ratios for all the test sensors, and the average compression ratio of the proposed method was also 3% lower than that of MPEG-4 ALS in fixed mode.

### 4.2. Processing Time

To compare the processing time of the proposed methods with those of other standard codecs with different modes, as described in Table 2, the encoding time of each compression method was measured by conducting the encoding process on a 3.2 GHz i5-4460 CPU with 8 GB of memory.

Table 4 summarizes the experimental results. As shown in the table, FLAC in fast mode showed the lowest encoding processing time, followed by WavPack in fast mode, MPEG-4 ALS with adaptive LPC order, and the proposed method. In order to investigate why the processing time of the proposed method was greater than that of the other codec, as shown in Table 4, we decomposed the encoding processing time for each processing block of the proposed method. In other words, the encoder of the proposed method was split into QMF analysis, LPC analysis for each sub-band, and entropy coding for each sub-band. Table 5 shows the processing time of each block according to different sensors. As shown in this table, the processing time for QMF analysis of the proposed method occupied approximately 56% of all the processing time. Thus, the sum of the processing times for LPC analysis and entropy coding was in between those of MPEG-4 ALS (Fixed) and MPEG-4 (Adaptive) since they did not have any QMF analysis. In addition, by comparing the processing times of LPC analysis in the low and high sub-bands, it was shown that the encoding processing time was increased according to the LPC order. This implied that it was necessary to adjust the LPC order for each sub-band by trading off the compression ratio and the processing time.

### 4.3. Analysis of Distortion

To examine how much the sensor signal was distorted due to compression, we measured the distortion between the original signal and the reconstructed signal. Generally, the operator of a surveillance system manually observes the presence of acoustic signatures, such as tonal signals radiated from targets, on the LOFAR-gram to confirm the presence or absence of a target [32]. In particular, the surveillance system detects a frequency energy peak that exceeds a detection threshold on the LOFAR-gram. Since the LOFAR-gram visualizes acoustic sensor signals as a two-dimensional acoustic image, we decided to use the peak signal-to-noise ratio (PSNR) as a distortion measure. The PSNR was defined as follows [10]:

(6) $$\mathrm{PSNR}=20\log_{10}\frac{\underset{i}{\max}\left|P_{i}\right|}{RMSE},1\leq i\leq N\;(\mathrm{dB})\;$$

where N is the total number of pixels, $P_{i}$ is the *i*th pixel value of the original data, and the root mean square error (RMSE) is defined as $\sqrt{\frac{1}{N}\sum_{i=1}^{N}{\left(P_{i}-Q_{i}\right)}^{2}}$ when $Q_{i}$ is the *i*th pixel value of the reconstructed data. While the PSNR has no absolute meaning, it is known that a PSNR of 25 dB is sufficient for photos [10]. However, the PSNR value should exceed 30 dB for the side-scan sonar image used in an underwater surveillance system [33,34].

In addition to PSNR, the structural similarity (SSIM) index [35] is also measured. This is because different types of degradation applied to the same image result in the same RMSE [36,37], while PSNR is sometimes known to be bad for distinguishing the structural content of an image.

Table 6 shows the compression ratio, PSNR, and SSIM of the proposed method according to different sensors, where the PSNR and SSIM values were calculated by composing LOFAR-gram data of 1600 × 1800 pixels with values ranging from 0 to 1. As shown in the first column of the table, the proposed method provided a PSNR of 62.94 dB on average. This implies that the proposed method could reconstruct the compressed signal with negligible distortion. Next, the PSNR of the proposed method was compared with that of a simple compression method, referred to as the bit-depth reduction (from 16 to 8 bits) technique [9]. Consequently, it was shown that the bit-depth reduction method distorted signals at around 25 dB; thus, the proposed method achieved higher PSNRs than the bit-depth reduction method for all the sensors. Finally, the PSNRs of SHORTEN [25], which is a well-known near-lossless waveform compression method, were measured according to different lossy mode settings of SHORTEN. The maximum number of bits per sample in SHORTEN was set to 9 or 10 bits. From the table, it can be seen that the compression ratio of the proposed method was almost the same as that of SHORTEN in 10-bit maximum mode, and both methods also achieved similar PSNR and SSIM on average.

Next, the RMSE values were measured for the proposed method and lossy methods, including a simple bit-depth reduction method and two different lossy modes of SHORTEN. As shown in Table 7, the proposed method had a much smaller RMSE than the simple bit-depth reduction method, and it provided similar RMSEs to the lossy modes of SHORTEN.

**Figure 10:**
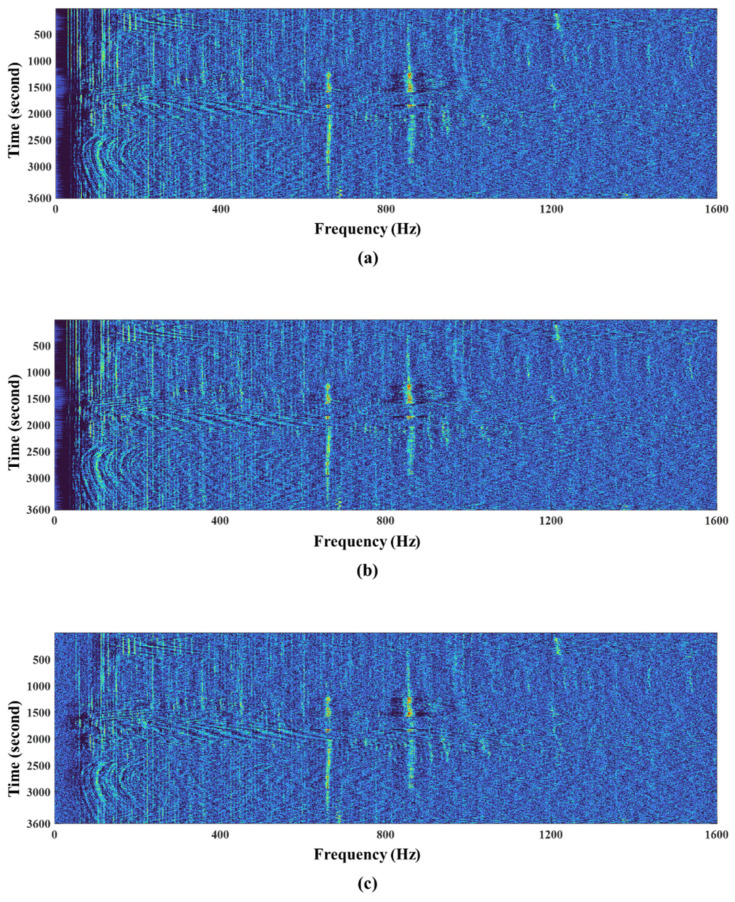

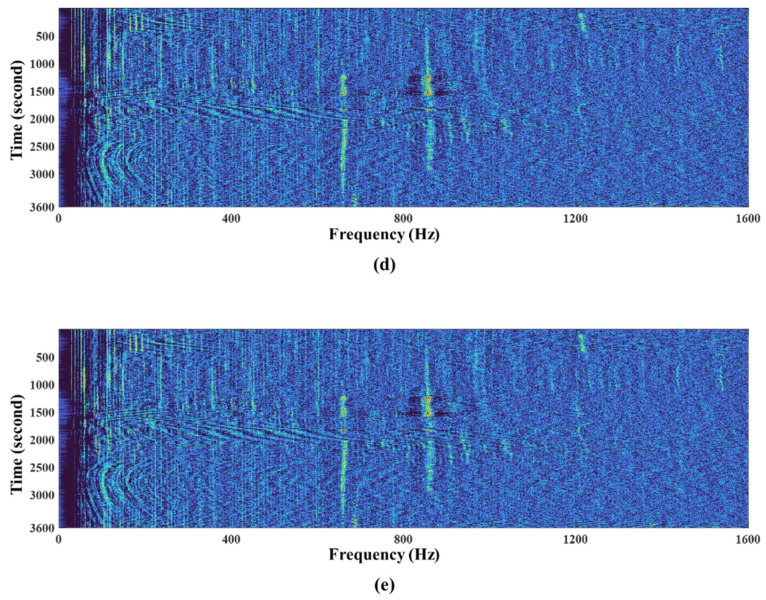
Illustration of LOFAR-grams of (**a**) an original sensor signal from sensor #6, (**b**) the reconstructed sensor signal by the proposed method, (**c**) the reconstructed sensor signal by the bit-depth reduction method, and (**d**,**e**) the reconstructed sensor signals by the lossy mode of SHORTEN with the maximum number of bits per sample was set between 9 and 10 bits, respectively.

Figure 10 illustrates the LOFAR-grams of the original sensor signal and the reconstructed signals by the proposed method, as well as the bit-depth reduction method. Compared with Figure 10a,b,d,e, there was almost no difference in all the frequency bands between the original signal and the reconstructed signal by the proposed method. However, the signal reconstructed by the bit-depth reduction method, as shown in Figure 10c, was distorted, especially in the low-frequency band, compared with the original signal.

## 5. Conclusions

This paper proposed a new low-complexity compression method of underwater acoustic sensor signals for underwater surveillance systems that was nearly lossless so that it could be customized for underwater acoustic sensor signals. The proposed method incorporated the concept of sub-band filter bank-based sub-band splitting into a scalable structure. In particular, both linear predictive coding and an entropy coding technique were used to reduce complexity and increase the compression ratio.

The performance of the proposed method was evaluated in terms of the compression ratios, processing time, and the degree of distortion by using three actual underwater sensors operating in coastal areas. Consequently, it was shown from the experiments that the proposed method achieved a higher compression ratio with a comparable processing time to popular or standardized lossless compression methods such as MPEG-4 ALS, FLAC, and WavPack. In addition, it was confirmed that the distortion of compressed acoustic sensor signals was negligible.

In future work, a machine-learning-based technique such as a deep belief network could be applied to find the optimal Rice parameter with the greatest influence on the entropy coding performance. Additionally, to further increase the compression ratio, we plan to apply a lossy compression technique to a high-frequency sub-band and more than two sub-bands for QMF analysis and synthesis.

## Figures and Tables

**Table 1:** Detailed information of acoustic sensor signals for performance evaluation.

Acquisition Sensor	Data Length	Sampling Rate	Resolution	Data Size
Sensor #4	1 h (3600 frames)	4096 Hz	16 bits	28.1 MB
Sensor #5				
Sensor #6				
Sensor #7				
Sensor #8				

**Table 2:** List of candidate codecs and their compression mode.

Notation	Codec	Compression Mode
A-1	MPEG-4 ALS (Fixed)	LPC order: 20 (Fixed), Rice encoding
A-2	MPEG-4 ALS (Adaptive)	LPC order: Adaptive, Rice encoding
A-3	MPEG-4 ALS (BGMC)	LPC order: 20 (Fixed), BGMC encoding
B-1	FLAC (Fast)	Fast mode (Fixed LPC Order)
B-2	FLAC (Best)	High-quality mode (maximum compression)
C-1	WavPack (Fast)	Fast mode
C-2	WavPack (HQ)	High-quality mode

**Table 3:** Comparison of the compression ratios (%) between the proposed method and other standard compression methods.

Sensor	Proposed	MPEG-4 ALS (Fixed)	MPEG-4 ALS (Adaptive)	MPEG-4 ALS (BGMC)	FLAC (Fast)	FLAC (Best)	WavPack (Fast)	WavPack (HQ)
#4	57.0	60.4	60.5	59.8	63.9	61.0	61.6	60.7
#5	59.7	63.1	63.1	62.5	66.5	63.6	64.4	63.3
#6	66.1	69.5	69.6	68.9	72.6	70.1	70.9	69.8
#7	68.4	71.7	71.8	71.2	75.3	72.3	73.4	72.0
#8	63.4	66.5	66.6	65.9	71.3	67.1	68.0	66.7
Avg.	62.9	66.2	66.3	65.7	69.9	66.8	67.7	66.5

**Table 4:** Comparison of the encoding processing time(s) of the proposed method with other codecs.

Sensor	Proposed	MPEG-4 ALS (Fixed)	MPEG-4 ALS (Adaptive)	MPEG-4 ALS (BGMC)	FLAC (Fast)	FLAC (Best)	WavPack (Fast)	WavPack (HQ)
#4	3.80	1.92	1.15	2.61	0.66	0.75	0.70	1.00
#5	3.68	1.92	1.17	2.61	0.55	0.66	0.74	1.05
#6	3.80	1.91	1.14	2.62	0.57	0.67	0.74	1.02
#7	3.70	1.92	1.15	2.64	0.54	0.66	0.73	1.04
#8	3.26	1.93	1.16	2.64	0.56	0.68	0.78	1.01
Avg.	3.65	1.92	1.15	2.62	0.58	0.68	0.74	1.02

**Table 5:** Encoding processing time(s) of each processing block of the proposed method.

Sensor	QMF Analysis	Low Sub-Band Encoding			High Sub-Band Encoding			SUM
		LPC Analysis	Entropy Coding	Etc.	LPC Analysis	Entropy Coding	Etc.	
#4	2.20	0.66	0.17	0.11	0.38	0.17	0.11	3.80
#5	2.00	0.69	0.18	0.14	0.33	0.22	0.12	3.68
#6	2.17	0.67	0.17	0.13	0.33	0.20	0.13	3.80
#7	2.06	0.66	0.17	0.15	0.34	0.20	0.12	3.70
#8	1.91	0.36	0.19	0.13	0.35	0.17	0.15	3.26
Avg.	2.07	0.61	0.18	0.13	0.35	0.19	0.13	3.65

**Table 6:** Comparison of the compression ratio, peak signal-to-noise ratios (PSNR), and structural similarity (SSIM) index between the original and processed acoustic sensor signals for the proposed method.

**Sensor**	**Proposed Method**			**Bit-Depth Reduction** **(from 16 to 8 bits)**		
	**Compression Ratio (%)**	**PSNR** **(dB)**	**SSIM**	**Compression Ratio (%)**	**PSNR** **(dB)**	**SSIM**
#4	57.01	43.81	0.9995	50.00	20.33	0.0663
#5	59.74	49.73	0.9994	50.00	26.04	0.6909
#6	66.13	50.69	0.9998	50.00	20.60	0.8520
#7	68.39	69.27	0.9998	50.00	30.60	0.8950
#8	63.44	50.58	0.9996	50.00	24.11	0.7283
Avg.	62.94	58.17	0.9996	50.00	25.21	0.6465
**Sensor**	**SHORTEN** **(Lossy Mode:** **Max. 9 Bits per Sample)**			**SHORTEN** **(Lossy Mode:** **Max. 10 Bits per Sample)**		
	**Compression Ratio (%)**	**PSNR** **(dB)**	**SSIM**	**Compression Ratio (%)**	**PSNR** **(dB)**	**SSIM**
#4	56.46	58.81	0.9987	61.66	67.33	0.9997
#5	56.74	45.88	0.9987	62.92	49.84	0.9994
#6	56.67	49.51	0.9989	62.92	48.48	0.9993
#7	56.74	54.58	0.9988	62.99	58.17	0.9995
#8	56.70	52.92	0.9985	61.64	61.72	0.9994
Avg.	56.66	53.43	0.9987	62.42	59.88	0.9995

**Table 7:** Comparison of root mean square error (RMSE) between the original and processed acoustic sensor signals for the proposed method.

Sensor	Proposed	Bit-Depth Reduction (from 16 to 8 bits)	SHORTEN (Lossy Mode: Max. 9 Bits per Sample)	SHORTEN (Lossy Mode: Max. 10 Bits per Sample)
#4	0.0064	0.0963	0.0011	0.0004
#5	0.0033	0.0499	0.0051	0.0032
#6	0.0029	0.0934	0.0033	0.0038
#7	0.0003	0.0295	0.0019	0.0012
#8	0.0030	0.0623	0.0023	0.0008
Avg.	0.0032	0.0663	0.0027	0.0019

## Data Availability

Part of the raw data analyzed in this study are available, with limited restriction, upon request from the corresponding author.

## References

[1] Sherman C.H., Butler J.L. (2007). Transducers and Arrays for Underwater Sound.

[2] Yin S., Ruffin P.B., Yu F.T.S. (2008). Fiber Optic Sensors.

[3] Waite A.D. (2002). Sonar for Practising Engineers.

[4] Hodges R.P. (2010). Underwater Acoustics: Analysis, Design and Performance of Sonar.

[5] Nielsen R.O. (1991). Sonar Signal Processing.

[6] Lv Z., Zhang J., Jin J., Li Q., Gao B. (2018). Energy consumption research of mobile data collection protocol for underwater nodes using an USV. Sensors.

[7] Hine R., Willcox S., Hine G., Richardson T. (2009). The wave glider: A wave-powered autonomous marine vehicle. Proceedings of the OCEANS 2009.

[8] Xiao L., Jiang D., Wan X., Su W., Tang Y. (2018). Anti-jamming underwater transmission with mobility and learning. IEEE Commun. Lett..

[9] Felis I., Martinez R., Ruiz P., Er-rachdi H. (2020). Compression techniques of underwater acoustic signals for real-time underwater noise monitoring. Proceedings.

[10] Salomon D. (2007). Data Compression: The Complete Reference.

[11] Bosi M., Goldberg R.E. (2002). Introduction to Digital Audio Coding and Standards.

[12] Johnson M., Partan J., Hurst T. (2013). Low complexity lossless compression of underwater sound recordings. J. Acoust. Soc. Am..

[13] Lixin L., Feng G., Jinqiu W. (2018). Underwater acoustic image encoding based on interest region and correlation coefficient. Complexity.

[14] Wong L.S., Allen G.E., Evans B.L. (2014). Sonar data compression using non-uniform quantization and noise shaping. Proceedings of the 48th Asilomar Conference on Signals, Systems and Computers.

[15] Burstein V., Henkel W. (2017). Linear predictive source coding for sonar data. Proceedings of the 2017 Global Conference on Signal and Information Processing (GlobalSIP).

[16] Kim Y.G., Jeon K.M., Kim Y., Choi C.-H., Kim H.K. (2017). A lossless compression method incorporating sensor fault detection for underwater acoustic sensor array. Int. J. Distrib. Sens. Netw..

[17] Shen S., Yang H., Yao X., Li J., Xu G., Sheng M. (2020). Ship type classification by convolutional neural networks with auditory-like mechanisms. Sensors.

[18] Domingos L.C.F., Santos P.E., Skelton P.S.M., Brinkworth R.S.A., Sammut K. (2022). A survey of underwater acoustic data classification methods using deep learning for shoreline surveillance. Sensors.

[19] Malvar H.S. (2007). Lossless and near-lossless audio compression using integer-reversible modulated lapped transforms. Proceedings of the 2007 Data Compression Conference.

[20] Knudsen V.O., Alford R.S., Emiling J.W. (1944). Survey of Underwater Sound, Report 3, Ambient Noise.

[21] Wenz G.M. (1962). Acoustic ambient noise in the ocean: Spectra and sources. J. Acoust. Soc. Am..

[22] Urick R.J. (1983). Principles of Underwater Sound.

[23] Agrawal S.K., Sahu O.P. (2013). Two-channel quadrature mirror filter bank: An overview. Int. Sch. Res. Notices.

[24] Ashida S., Kakemizu H., Nagahara M., Yamamoto Y. (2004). Sampled-data audio signal compression with Huffman coding. Proceedings of the SICE 2004 Annual Conference.

[25] Robinson T. (1994). SHORTEN: Simple Lossless and Near-Lossless Waveform Compression.

[26] Liebchen T., Reznik Y.A. (2004). MPEG-4 ALS: An emerging standard for lossless audio coding. Proceedings of the 2004 Data Compression Conference.

[27] Yesh P.S., Rice R.F., Miller W. (1991). On the Optimality of Code Options for a Universal Noiseless Coder.

[28] Donada F.S. (2020). On the optimal calculation of the Rice coding parameter. Algorithms.

[29] Shen H., Pan W.D., Dong Y., Jiang Z. (2017). Golomb-Rice coding parameter learning using deep belief network for hyperspectral image compression. Proceedings of the 2017 International Geoscience and Remote Sensing Symposium (IGARSS).

[30] Reznik Y.A. (2004). Coding of prediction residual in MPEG-4 standard for lossless audio coding (MPEG-4 ALS). Proceedings of the 2004 International Conference on Acoustics, Speech, and Signal Processing (ICASSP).

[31] (2007). Information Technology—Coding of Audio-Visual Objects—Part 5: Reference Software, Amendment 10: SSC, DST, ALS and SLS Reference Software.

[32] Wan C.R., Goh J.T., Chee H.T. (2000). Optimal tonal detectors based on the power spectrum. IEEE J. Ocean. Eng..

[33] Wu D., Du X., Wang K. (2018). An effective approach for underwater sonar image denoising based on sparse representation. Proceedings of the 2018 International Conference on Image, Vision and Computing (ICIVC).

[34] Luo J., Liu H., Huang C., Gu J., Xie S., Li H. (2013). Denoising and tracking of sonar video imagery for underwater security monitoring systems. Proceedings of the 2013 International Conference on Robotics and Biomimetics (ROBIO).

[35] Wang Z., Bovik A.C., Sheikh H.R., Simoncelli E.P. (2004). Image quality assessment: From error measurement to structural similarity. IEEE Trans. Image Process..

[36] Horé A., Ziou D. (2010). Image quality metrics: PSNR vs. SSIM. Proceedings of the 2010 International Conference on Pattern Recognition.

[37] Wang Z., Bovik A.C. (2009). Mean squared error: Love it or leave it? A new look at signal fidelity measures. IEEE Signal Process. Mag..

